# Reactive oxygen species may be involved in the distinctive biological effects of different doses of ^12^C^6+^ ion beams on *Arabidopsis*

**DOI:** 10.3389/fpls.2023.1337640

**Published:** 2024-01-19

**Authors:** Yue Yin, Dongjie Cui, Qing Chi, Hangbo Xu, Panfeng Guan, Hanfeng Zhang, Tao Jiao, Xiaojie Wang, Lin Wang, Hao Sun

**Affiliations:** ^1^ Henan Key Laboratory of Ion-beam Bioengineering, School of Physics and Microelectronics, Zhengzhou University, Zhengzhou, China; ^2^ State Key Laboratory of Cotton Biology, School of Agricultural Sciences, Zhengzhou University, Zhengzhou, China; ^3^ Sanya Institute, Zhengzhou University, Zhengzhou, China; ^4^ Asset Management Co., Ltd, Henan Institute of Science and Technology, Xinxiang, China; ^5^ School of Bioengineering, Xinxiang University, Xinxiang, China; ^6^ College of Biology and Food Engineering, Anyang Institute of Technology, Anyang, China

**Keywords:** heavy ion beam, *Arabidopsis thaliana*, contemporary biological effects, plant growth, RNA-seq

## Abstract

**Introduction:**

Heavy ion beam is a novel approach for crop mutagenesis with the advantage of high energy transfer line density and low repair effect after injury, however, little investigation on the biological effect on plant was performed. 50 Gy irradiation significantly stimulated the growth of Arabidopsis seedlings, as indicated by an increase in root and biomass, while 200 Gy irradiation significantly inhibited the growth of seedlings, causing a visible decrease in plant growth.

**Methods:**

The Arabidopsis seeds were irradiated by 12C6+. Monte Carlo simulations were used to calculate the damage to seeds and particle trajectories by ion implantation. The seed epidermis received SEM detection and changes in its organic composition were detected using FTIR. Evidence of ROS and antioxidant systems were analyzed. RNA-seq and qPCR were used to detect changes in seedling transcript levels.

**Results and discussion:**

Monte Carlo simulations revealed that high-dose irradiation causes various damage. Evidence of ROS and antioxidant systems implies that the emergence of phenotypes in plant cells may be associated with oxidative stress. Transcriptomic analysis of the seedlings demonstrated that 170 DEGs were present in the 50 Gy and 200 Gy groups and GO enrichment indicated that they were mainly associated with stress resistance and cell wall homeostasis. Further GO enrichment of DEGs unique to 50 Gy and 200 Gy revealed 58 50Gy-exclusive DEGs were enriched in response to oxidative stress and jasmonic acid entries, while 435 200 Gy-exclusive DEGs were enriched in relation to oxidative stress, organic cyclic compounds, and salicylic acid. This investigation advances our insight into the biological effects of heavy ion irradiation and the underlying mechanisms.

## Introduction

Heavy ions are positively charged particles with an atomic number of two or more, which are typically missing some or all of their outer electrons. These ions can be found in small amounts in space and are a component of space radiation. With advances in nuclear technology and the development of hardware such as accelerators, researchers are now able to generate heavy-ion beams at precise energy levels. One of the most prominent physical properties of heavy-ion beams is the Bragg curve, which is an inverted depth dose distribution compared to conventional radiation (Kraft, 2000; Hasson et al., 2013). Recently, the heavy-ion beams have been used as a novel effective mean of mutagenesis in plant and microbial breeding (Tanaka et al., 2010). The heavy-ion beams deposit more energy and have a higher linear energy transfer (LET) than low LET radiations such as X-rays or gamma-rays, making them more difficult to be repaired by cells (Hirano et al., 2015). As a result, the heavy-ion beams can induce stronger biological effects than other forms of radiation.

In general, most studies concentrate on the damage effect of heavy ion beam irradiation. At the cellular level, the damage included the thinning and perforation of the cell wall, rupture of the cell membrane and organelles, and cell lysis and death (Vilaithong et al., 2000; Yu et al., 2002). At the molecular level, the heavy ion beam irradiation could cause the breaks, cross-links, and high-level structural changes of DNA double strand, as well as single-base substitutions, insertions, and deletions (Prise et al., 2001; Hada and Georgakilas, 2008; Ritter and Durante, 2010). Furthermore, ion beam irradiation could also induce changes at the epigenetic levels, such as DNA methylation (Qian et al., 2010; Yu et al., 2011; Lima et al., 2014). The transcriptional and proteomic response of plants can differ due to the variety of plants and ion type (Xiong et al., 2020; Li et al., 2022).

Reactive oxygen species (ROS) was associated with the plant growth and may influence the response of plants to radiation (Huang et al., 2019; Tan et al., 2023). For example, ROS production in mitochondria has been observed under radiation including gamma (Yoshida et al., 2012; Kawamura et al., 2018). Previous studies have shown that different doses of irradiation lead to changes in ROS and antioxidant gene expression in plants. Zhang et al. (2008) found that ^12^C^6+^ irradiation of wheat at 10-80 Gy all caused changes in seedling ROS levels, but 20 Gy ROS levels were the highest; Wang et al. (2018a) found that irradiation of *Arabidopsis thaliana* seeds with 50-200 Gy ^12^C^6+^ ion beams all resulted in the accumulation of ROS in the seedlings and the expression of antioxidant enzyme systems was also increased. However, the transcriptomic response of plants under different dose of heavy ions irradiation remains unclear. Given that ROS induced by ionizing radiation have a very important role in the biological effects of heavy ion beam irradiation, we examined several ROS-related metrics in seedlings and analyzed them jointly with comparative transcriptome results.

Herein, this study investigated the effects of different dose of heavy ion beam irradiation on *Arabidopsis* by algorithmically simulating the damage patterns produced by heavy ion beam irradiation on seeds, combined with the comprehensive analysis of seed morphology, as well as the seedling phenotype, physiology and transcriptome. This study provides a theoretical basis for selecting doses in heavy ion beam mutagenesis breeding work. Additionally, it suggests an idea for investigating the biological effects of plants in response to varying doses of heavy ion beam irradiation.

## Materials and methods

### Irradiation and Monte Carlo simulation

*Arabidopsis* (cv. ‘Columbia’) seeds were placed in petri dishes with a diameter of 35 mm, a depth of 10 mm and a thickness of 1 mm. These seeds were then exposed to ionizing radiation using the shallow treatment and biological irradiation terminal (TR4) of the Heavy Ion Research Facility in Lanzhou (HIRFL) at the Institute of Modern Physics, Chinese Academy of Sciences (IMP-CAS). The irradiation condition was air environment, carbon ions with an initial energy of 80 MeV/u, a temperature of 12 ± 2°C, a beam current intensity of 30 nA, a dose rate of 80 Gy/min, and doses of 50 and 200 Gy.

Monte Carlo methods are employed to investigate the ion implantation processes. Monte Carlo simulations were conducted to simulate the interaction between 80 MeV/u carbon ions (^12^C^6+^) and *Arabidopsis* seeds by utilizing the TRIM program of the SRIM (Ions in Matter stop range of Ions in Matter) software (Ziegler et al., 2010). Since the seeds were not included in the built-in parameters of SRIM, the necessary parameters were manually calculated. To determine the density, the weight of seeds in a 35 mm petri dish was determined and the average density was 0.7203 g/cm^3^. The content of other components (proteins, lipids, sugars, etc.) of *Arabidopsis* seeds was obtained from the literature (**Supplementary Table S1**). Petri dish made of polystyrene plastic from Corning were represented in the model (**Supplementary Figure S1**). The ‘detailed calculation with full damage cascades’ mode was selected regarding type of SRIM calculation.

### Scanning electron microscopy and fourier transform infrared spectrometer spectra analysis

*Arabidopsis* seeds used for SEM and FTIR analysis were gradient dehydrated with ethanol (40%, 60%, 80%, and 100%). After being glued to a sample stage with conductive tape (Nisshin EM), the seeds were sprayed with Pt and observed using a Hitachi SU3500 scanning electron microscope for SEM analysis. For FTIR analysis, the seeds were ground and mixed with potassium bromide, which was tested in the wavenumber region of 500-4000 cm^-1^ using a FTIR spectrometer (Tensor, Bruker, Germany). Each sample was replicated three times for biological accuracy.

### Plant morphological observation

After being sterilized in a disinfector (30% H_2_O_2_: 80% ethanol = 1:3; v/v) for 1 min, *Arabidopsis* seeds were transferred to dry filter paper with a 1 mL pipette. The seeds were then vernalized at low temperature (4°C) for 48 h and planted in 90 mm × 10 mm sterile petri dishes containing 1/2 MS (Murashige Skoog Medium). The petri dishes were then placed vertically and placed in a growth chamber at 22 ± 2 °C with a light of 5000 lx and a 14/10 h light-dark cycle. The seedlings were grown in 1/2 MS for 7 days and the plant height and root length were measured with Image J (Rawak Software, Inc. Germany). Afterwards, the seedlings were collected and dried for 24 h at 45°C. The final dry weight was then measured. Thirty seedlings from each group were selected with 3 replications.

### Microscopic observation of root tip

Root tip of *Arabidopsis* was stained with PI as Benfey described (Long et al., 2010; Sozzani et al., 2010). Briefly, the whole seedling root samples were immersed in 10 μg/mL propidium iodide (PI) solution for a period of 1 to 10 min and then observed under a confocal microscope (Zeiss LSM880).

### Determination of ROS and malondialdehyde

The detection of superoxide radical (·O_2_^-^) content was based on the method proposed by (Elstner and Heupel, 1976) with slight modifications. The 0.2 g of seedlings were homogenized in 1 mL of 50 mM phosphate buffer (pH=7.8) and then centrifuged at 12000 rpm at 4°C for 10 min. Then, 0.6 mL of the supernatant was mixed with 0.1 mL of 10 mM hydroxylamine hydrochloride and 0.5 mL of 65 mM phosphate buffer (pH=7.8) and then left to rest at 25°C for 1 h. After centrifugation at 12000 g at 4°C for 10 min, 1 mL of 17 mM p-aminobenzenesulfonic acid and 1 mL of 7 mM α-naphthylamine were added and allowed to stand for 20 min at 25°C. The absorbance was then measured at 530 nm.

The rate of hydroxyl radical (·OH) production was measured by oxidizing bromopyrogallol red (BPR) (Wang et al., 2018a). The 2 g of 7-day-old seedlings were homogenized in 1.5 mL of deionized water, which was then centrifuged at 5000 g at 4°C for 10 min. Subsequently, 1 mL of the supernatant was added with 0.3 mL of 1% (v/v) H_2_O_2_, 0.3 mL of 1 mM BPR solution and 0.2 mM FeSO_4_ solution. The absorbance was detected at 550 nm wavelength.

H_2_O_2_ content was determined by Patterson’s method (Patterson et al., 1984). The 0.2 g of 7-day-old seedlings were homogenized in 5 mL of pre-cooled acetone and centrifuged at 10,000 g at 4°C for 15 min. Subsequently, 0.2 mL of concentrated ammonia and 0.1 mL of 20% TiCl_4_ were added to 1 mL of the supernatant, mixed gently and reacted at 25°C for 5 min before centrifuging at 8000 g at 4°C for 10 min. The precipitate was then rinsed repeatedly with pre-cooled acetone and then 1 mL of 1 M H_2_SO_4_ was added to dissolve it, after which the absorbance was assayed at 410 nm.

Malondialdehyde (MDA) content was determined as described in a previous study (Qi et al., 2015). The 0.2 g seedlings were homogenized in 5 mL of 10% (w/v) trichloroacetic acid and centrifuged at 5000 g for 10 min at 4°C. Subsequently, 2 mL of the supernatant was blended with 2 mL of 0.6% thiobarbituric acid (TBA) [dissolved in 10% trichloroacetic acid (TCA)]. This homogenate was then incubated at 100°C for 30 min and centrifuged at 5000 g for 10 min. The supernatant was used to measure the absorbance value with a spectrophotometer at 450, 532 and 600 nm.

### Determination of antioxidant systems

About 0.2 g 7-day-old seedlings were homogenized in 2 mL of 50 mM phosphate buffer containing 4% polyvinylpyrrolidone (PVP) and 5 mM dithiothreitol (DTT) and centrifuged at 12,000 g for 15 min at 4°C. The supernatant enzyme extraction solution (EES) was used for the determination of superoxide dismutase (SOD), peroxidase (POD), and catalase (CAT) activity.

The SOD activity was determined by monitoring the inhibition of the photochemical reduction of nitroblue tetrazolium (Giannopolitis and Ries, 1977). Each 3 mL reaction mixture was made up of 50 mM phosphate buffer (pH=7.8), 0.1 mM EDTA, 130 mM methionine, 0.75 mM NBT, 0.02 mM riboflavin, and 0.1 mL EES. The reaction mixture was illuminated for 15 min at a light intensity of 5000 lx. The mixture with no light application was used as a control. The absorbance was recorded at 560 nm. The quantity of enzyme required to reduce the NBT by 50% was used to calculate the SOD activity, which was measured in units.

The POD activity was measured using guaiacol (Zhang and Kirkham, 1994). The 0.2 mL of guaiacol solution and 0.2 mL of EES were added to 2.5 mL of phosphate buffer (pH=7, 50 mM), and then 0.1 mL of H_2_O_2_ was further added to detect the change in absorbance at 470 nm within 3 min.

To gauge CAT activity, the approach of Cakmak et al. (1992) was employed. This involved in the blending of 0.1 mL of 50 mM phosphate buffer (pH=7.0) and 2.9 mL of 20 mM H_2_O_2_ with 0.1 mL of EES. The absorbance value of the reaction mixture was determined at 240 nm for a period of 3 min.

Ascorbic acid (AsA) concentration were measured according to the method of Chen et al. (2015). 0.2 g of 7-day-old seedlings were homogenized by adding 2 mL of 0.2 M HCl at 4°C, followed by centrifugation at 5000 g at 4°C for 15 min. Subsequently, 0.5 mL of supernatant was blended with 0.4 mL of 0.2 M NaOH and 50 μL of 0.2 M NaH_2_PO_4_, and then the absorbance was measured at 265 nm.

### RNA extraction, transcriptome sequencing and bioinformatics analysis

Seedlings from each group (Col, 50 Gy, and 200 Gy) were cultured for 7 days and used for transcriptome sequencing. The mRNA was extracted with TRIZOL and sequenced using the Illumina Hiseq 4000 platform. The raw reads were filtered for low-quality data, contamination, and high N content. HISAT (Kim et al., 2015) was used to map the clean reads to the reference genome (Lamesch et al., 2012). GATK (McKenna et al., 2010) was then used to detect single nucleotide polymorphism (SNP) and insertion/deletion (INDEL). The transcript with protein coding potential was added to the reference gene sequence to form a complete reference sequence. Bowtie2 (Langmead and Salzberg, 2012) was used to map the clean reads to the reference sequence. RSEM (Li and Dewey, 2011) was used to calculate the expression levels of genes and transcripts. NOIseq algorithm (Tarazona et al., 2015) was used to detect DEGs. TBtools (Chen et al., 2020) was used to visualize the gene expression levels. Gene Ontology (GO) enrichment analysis was performed using TopGO, where the list of genes and number of genes in each term were calculated using the GO term annotated DEGs. The *P*-value was calculated by hypergeometric distribution (*P*- value < 0.05 indicating significance of enrichment) to identify the GO terms that were significantly enriched by the DEGs compared to the whole genomic background, thereby determining the main biological functions of DEGs.

### Gene expression analysis using quantitative real time PCR

Total mRNA prepared for RNA-seq was also subjected to quantitative real-time polymerase chain reaction (qRT-PCR) analysis. Reverse Transcriptase M-MLV (Takara, Japan) was used to generate cDNA for qRT-PCR analysis. SYBR® Premix Ex Taq™ (Takara, Japan) was employed on a Mastercycler® ep realplex Real-time PCR System (Eppendorf, German) to assess expression levels. The 2^-ΔΔCt^ method was used to calculate the fold changes in expression. Tubulin beta-1 chain (AT1G75780) was used as the internal control, and the primers used were listed in **Supplementary Table S3**. The experiments were conducted three times. RNA-seq data was uploaded in Genome Sequence Archive (GSA, https://ngdc.cncb.ac.cn/gsa/), accession number: CRA013460.

## Results and discussion

### Monte Carlo simulation of ion beam implantation

**Figure 1:**
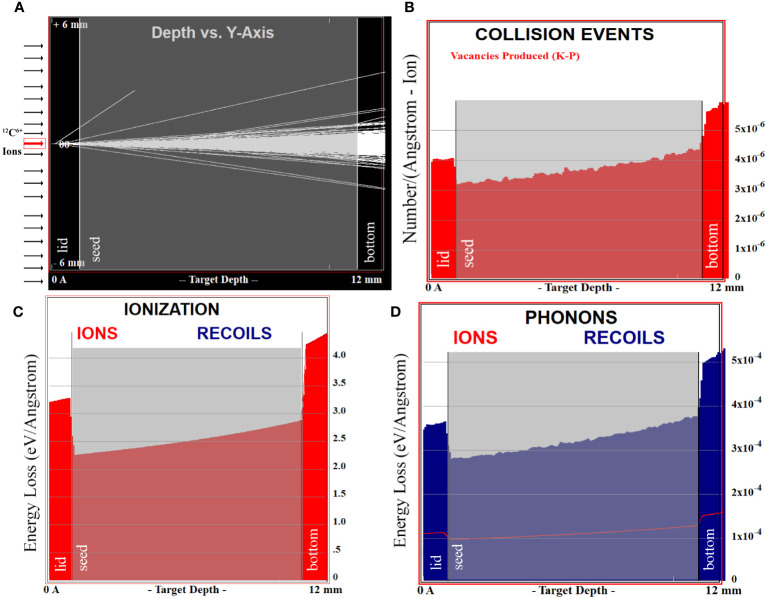
Simulation results of the TRIM program. In **(A–D)**, the target will be divided into three layers, the lid, the bottom and the middle layer of seeds (as shown in gray pattern). **(A)** represents the trajectory of ions through the target. **(B)** demonstrates the vacancies in the target caused by the incident ions. **(C, D)** show the ionization energy loss and phonon energy loss of the injected ion.

Irradiation damage and depth was simulated using the TRIM program in the SRIM software. The simulation results showed that the penetration depth of irradiation was 24.44 mm, which was significantly larger than the depth of the dish (10 mm) under irradiation. In addition, the energy deposition and the level of damage increased slightly with the increase of target depth (**Figures 1A–D**).

The charged particles could decelerate and transfer energy to the medium as they enter it, resulting in a sharp energy release at the end of range, which was known as the Bragg peak of a heavy ion beam (Blakely et al., 1984). However, since our targets were not thick enough to reach the end of the range, the damage was uniform across the target. The interaction between charged particles and target matter can be divided into four types of collisions, namely inelastic collisions with electrons outside the nucleus (Ionization loss), elastic collisions with the nucleus, inelastic collisions with the nucleus (Radiation loss), and elastic collisions with electrons outside the nucleus. Under the condition of high-energy ions, the ionization loss far exceeded radiation loss, about 2000:1 (Lindhard et al., 1963). The inelastic collisions between charged particles and extra-nuclear electrons were the primary mode of energy loss, which was consistent with our simulation results. Analysis of the data showed that 99.96% of the damage was caused by the ionization of incident ions, while 0.03% was attributed to the ionization of the recoil atoms and the other remaining 0.01% was diffused by the recoil atoms in the form of phonons (**Figure 1D**).

In high-energy ion beam irradiation, a higher initial energy often corresponds to a lower linear energy transfer (LET) within a specific range, reducing damage to ion tracks (Berger et al., 2006; Kazama et al., 2008; Kazama et al., 2011). Despite the ^12^C^6+^ ion beam in our study having a non-fatal LET, the elevated irradiation dose still induced significant biological effects. **Figure 1A** stimulated the results of 9999 ions from a single channel, where most of the ions were found to pass through the target. The Monte Carlo algorithm was used to simulate the irradiation process, which revealed the occurrence of rare events such as the collision of ^12^C^6+^ ions with the nucleus of the target atoms, resulting in a sharp deceleration of the trajectory and substantial damage at the end of the range. As the irradiation dose correlated with the injected ion count, higher doses required more injected ions, elevating the probability of nuclear collisions. This might explain why high doses of high-energy injected ions could cause severe target damage despite the majority ions passing through.

### Morphology observation of seeds after ion beam irradiation

The physical effects of particle radiation have been extensively researched. The extent of damage was dependent on the type of particle and plant. Studies in the condensed matter physics have shown the that irradiation can cause holes in materials (Miyoshi, 1996; Das and Rao, 2021). Wang and Wang (2013) investigated the effects of N^+^ irradiation on the wheat seeds and found that higher doses of irradiation caused etching and holes in the seed coat. Similarly, when *Arabidopsis* was irradiated with low-energy N^+^, abrasion-like damage was observed in the columella and radial primary wall of the seed coat cells, accompanied by a significant reduction in their thickness (Wang et al., 2004). Li et al. (2005) studied the effects of irradiation on *Cedrus deodara* pollen using SEM and atomic force microscopy. The result showed that the pollen was more sensitive to irradiation than the seed. Even a very low dose irradiation could cause significant damage to the pollen’s structure.

**Figure 2:**
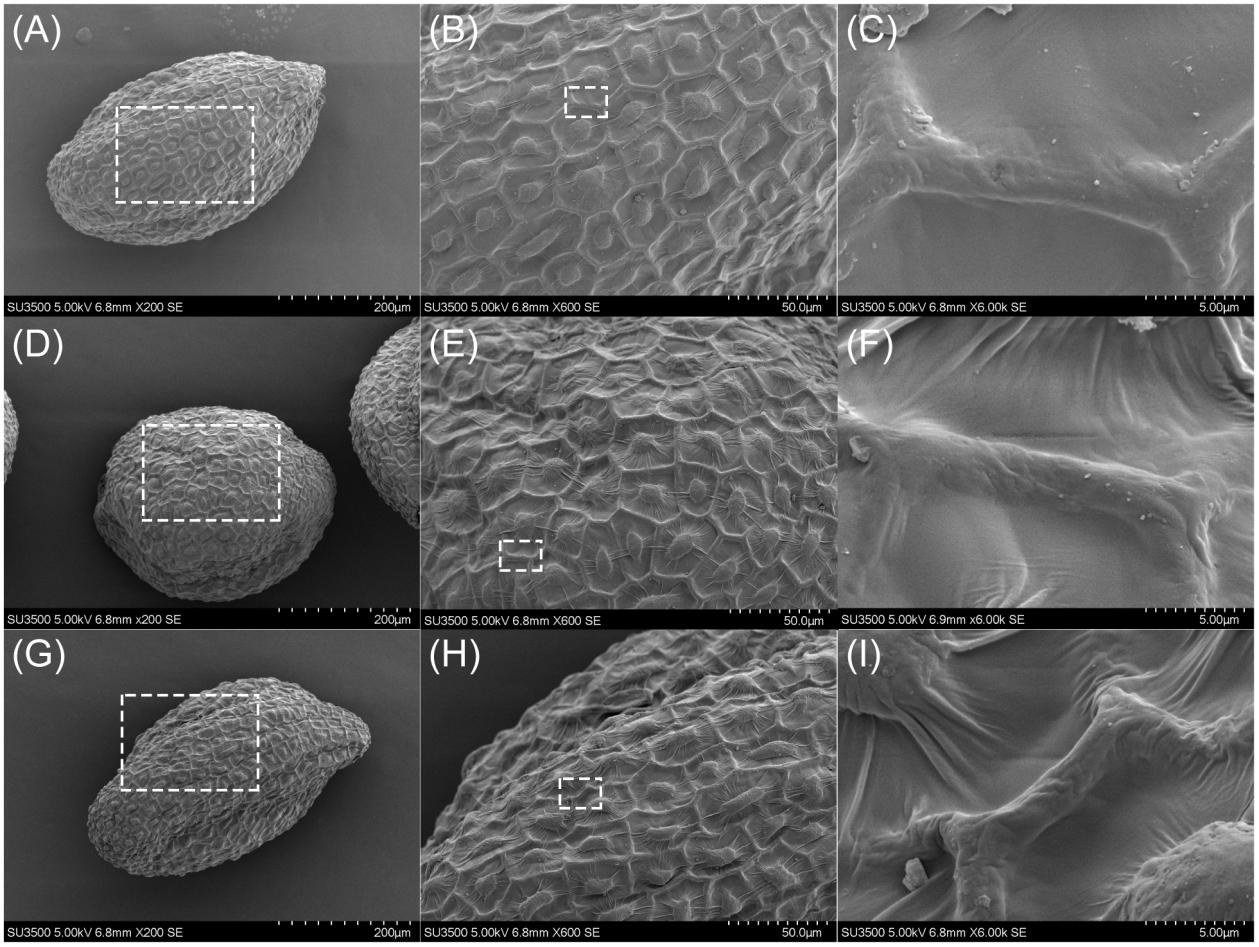
SEM observations of *Arabidopsis* seeds irradiated by ^12^C^6+^ ion beam. **(A–C)** show the results of Col seeds without irradiation observed at 200x, 600x and 6000x, respectively, and similarly, **(D–F)** and **(G–I)** show the results of the observations at 50 Gy and 200 Gy. **(B, E)** and **(H)** are the amplification of the insets in **(A, D)** and **(G, C, F, I)** are the amplification of the insets in **(B, E, H)** respectively.

However, there was no visible damage observed in our study (**Figure 2**). Upon irradiation, *Arabidopsis* seeds merely exhibited wrinkling of the seed coat cells, which worsened with the increase of irradiation dose. Even at 200 Gy, magnified inspection failed to detect any holes in the seed coat, making it difficult to distinguish irradiated seeds from non-irradiated seeds (**Figures 2D–I**). This was consistent with the study of Iwai et al. (1999) by using ^14^N^+^ to irradiate leaf disks of *Nicotiana plumbaginifolia*. Our Monte Carlo simulations revealed that the depth of injection was far greater than the thickness of the target, yet no visible damage was detected on the seed coats. These results demonstrated that the biological effects of ion beam irradiation on *Arabidopsis* seeds were mainly induced by ‘ionization loss’ rather than the high energy release events at Bragg Peak.

### FTIR investigation

**Figure 3:**
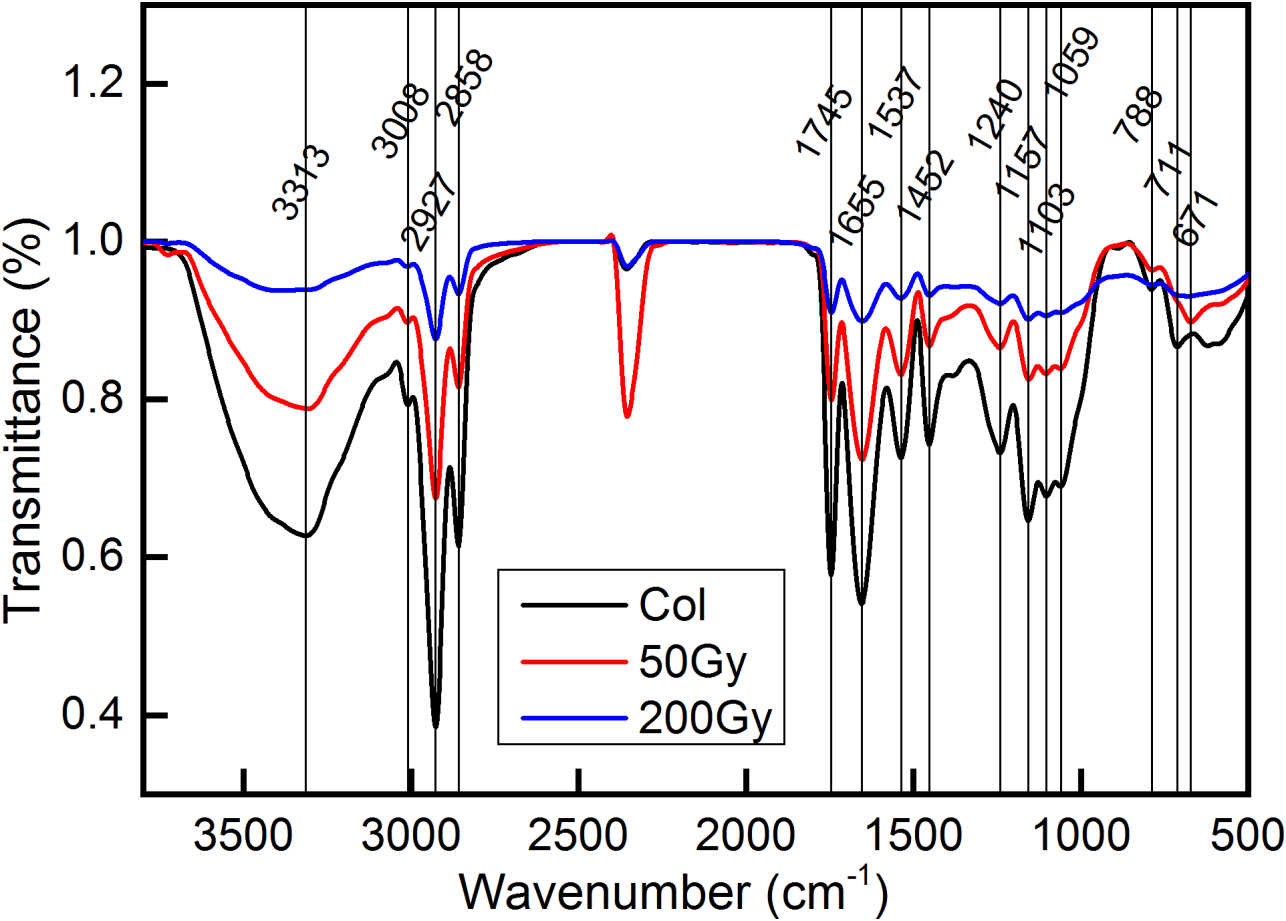
FTIR detection of *Arabidopsis* seeds irradiated by ^12^C^6+^ ion beam. The detection wave number range was 500 - 4000 cm^−1^. The red line represents the transmittance of *Arabidopsis* seeds after 50 Gy irradiation, blue represents that after 200 Gy irradiation and black line represent the control.

FTIR spectroscopy was employed to analyze the biomolecules in *Arabidopsis* seeds (**Figure 3**). After filtering out the data related to N-H and O-H stretching of 4000-3770 cm^-1^, environmental CO_2_ of 2800-1800 cm^-1^, and high noise ratio data below 800 cm^-1^ (Duygu et al., 2009; Kelly et al., 2011), a total of 12 characteristic peaks were identified. These peaks were located at 3313, 3008, 2927, 2858, 1745, 1655, 1537, 1452, 1240, 1157, 1103, and 1058 cm^-1^, respectively. These peaks mainly correspond to macromolecules with amide bonds in protein and phosphate bonds in nucleic acid (Byler and Susi, 1986; Movasaghi et al., 2008; Hu et al., 2017; Sharma et al., 2019).Previous studies have shown that the infrared absorption by biomolecules mainly occurred in the 3050-2800 cm^-1^ and 1800-900 cm^-1^, which was known as the CH stretch region and the biochemical fingerprint region (Huleihel et al., 2002). The CH stretch region mainly represented the stretching vibration of hydrocarbon bonds, which was highly correlated with lipid and hydrocarbon structures. The biochemical fingerprint region mainly reflected a variety of biomolecules, including lipid-related C=O (1600-1800 cm^-1^), protein secondary structure-related amide I (1603-1700 cm^-1^), amide II (1480-1560 cm^-1^), amide III (1180-1300 cm^-1^), amino acid-related COO- (around 1400 cm^-1^), nucleic acid-related PO_2_^-^ (1040-1100, 1220, 1240 cm^-1^), sugar synthesis-related C-OH (1030 cm^-1^), and a characteristic peak for H_2_O_2_ near 3309 cm^-1^ (Van de Voort et al., 1994; Fabian et al., 1995; Chiriboga et al., 1998; Eckel et al., 2001; Shetty et al., 2006; Movasaghi et al., 2008; Baker et al., 2014).

As shown in **Figure 3**, there was no obvious distinction in peak positions among the different treatment groups. The reason may be that the irradiation injuries in this study were largely caused by the ionization loss (**Figure 1**), thereby reducing the occurrence of radiation loss. As a result, the probability of incident ions colliding with atoms in the target was minimal, and the likelihood of chemical bond damage yielding new matter was negligible.

### Effect of irradiation on *Arabidopsis* seedlings growth and development

**Figure 4:**
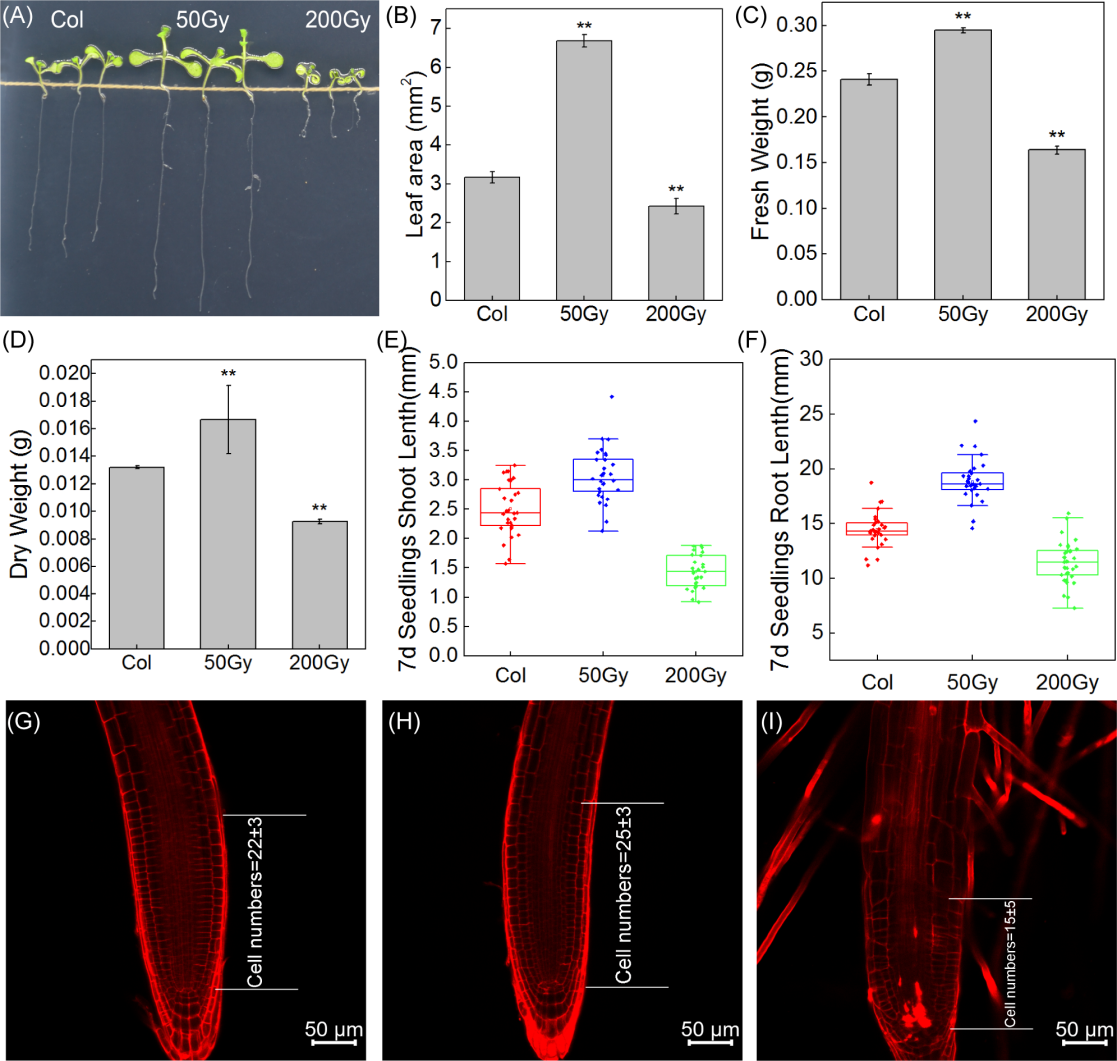
Observation of *Arabidopsis* seedlings from irradiated seeds. **(A)** Photographs of the three groups of 7-day-old seedlings; **(B)** Leaf area of the seedlings, 10 cotyledons were randomly selected; The fresh weight **(C)** and dry weight **(D)** of *Arabidopsis* seedlings were weighed, Data are means ± SE (n=3); **(E, F)** The shoot and root length of 7-day-old seedlings, Data are means ± SE (n=30); Asterisks indicate significant differences (* for *P* < 0.05, ** for *P* < 0.01); **(G–I)** Confocal microscopic observation of root tip.

The wild-type *Arabidopsis* seeds were exposed to different doses of carbon ion beams irradiation to determine the biological response of irradiated seedlings. Leaf area almost doubled at 50 Gy compared to control, while it decreased by 18% at 200 Gy (**Figure 4**). Meanwhile, fresh and dry weight increased by 16% and 23%, respectively, with 50 Gy and decreased by 34% and 31% with 200 Gy irradiation compared to Col (**Figures 4C, D**). The comparison of root length and shoot length can also intuitively show the difference in the growth state of seedlings. It can be clearly seen from the figure that both root and shoot length are significantly increased in 50 Gy compared with the control, while the development of 200 Gy is limited (**Figures 4A, E, F**). The results indicate that 50 Gy ^12^C^6+^ irradiation could promote the growth of *Arabidopsis thaliana* at the seedling stage. Conversely, 200 Gy ^12^C^6+^ irradiation was observed to inhibit the growth of *Arabidopsis* seedlings.

The microscopic observation of the root tips of *Arabidopsis* seedlings revealed that the number of cells in the meristematic zone of the root tip was slightly increased after 50 Gy irradiation treatment, indicating that the low-dose irradiation stimulated the division of stem cells and facilitated the production of differentiated cells, thus promoting root elongation (**Figures 4G, H**). In contrast, the 200 Gy irradiation caused much more serious damage to the growth and development of *Arabidopsis* (**Figure 4I**). Moreover, the root tips exhibited an irregular curved shape and a reduced differentiation zone.

### Redox regulation of *Arabidopsis* response to ion beam irradiation

The ionizing radiation can induce direct and indirect biological effects on the living organisms. The direct effects involve in the absorption of energy by biological molecules, causing damage to macromolecules. Indirect effects are resulted by the radiolysis of water, which produces various of free radicals or ROS. The growth promotion phenomenon caused by low-dose ion beam irradiation has not been fully explained, although some studies suggest that it may be related to the small amount of ROS generated by irradiation (Shi et al., 2011). In plants, the ·O_2_^-^ is converted to H_2_O_2_ under the catalysis of SOD. H_2_O_2_ is then degraded by CAT, POD, AsA and other substances. Although H_2_O_2_ is an essential signaling molecule in plants, excessive production of ·O_2_^-^ can lead to the formation of the highly destructive ·OH. The ·OH is the most damaging, as it diffuses quickly and reacts with intracellular biomolecules at a rapid rate, leading to the rupture of their structure, which is one of the main causes of radiation-induced DNA strand breaks.

**Figure 5:**
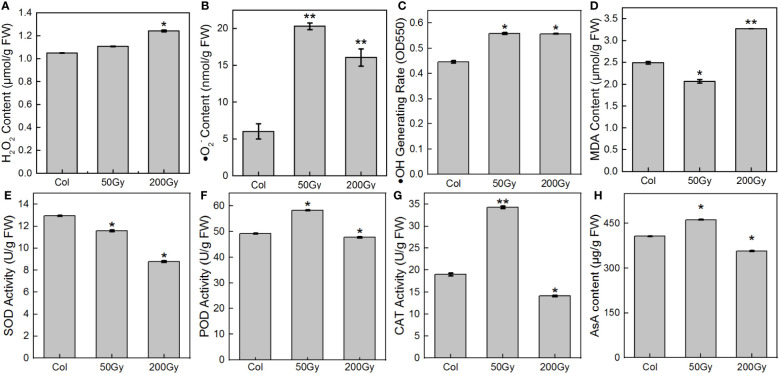
ROS and antioxidant system assays in 7-day-old seedlings. **(A)** H_2_O_2_ content; **(B)** ·O_2_^-^ content; **(C)** ·OH production rate; **(D)** MDA content; **(E)** SOD activity; **(F)** POD activity; **(G)** CAT activity; **(H)** AsA content. (* for *P* < 0.05, ** for *P* < 0.01).

It has been widely established that ROS are powerful oxidizers and plays major roles in plant signaling, thereby regulating plant growth and development (Chen et al., 2015; Tan et al., 2023). Upon exposure to 50 Gy irradiation, ROS levels, particularly ·O_2_^-^, were significantly elevated compared to the control (**Figure 5B**). The level of MDA (**Figure 5D**), an indicator of membrane lipid peroxidation, was reduced, indicating that the plants were not significantly damaged and even benefited from the irradiation. In contrast, when exposed to 200 Gy, H_2_O_2_ levels were significantly higher than those of the 50 Gy dose and the control, while ·O_2_^-^ levels were considerably lower than the 50 Gy dose, but still higher than the control (**Figure 5**). Furthermore, MDA levels were significantly higher than the control, demonstrating that the plants were severely damaged. Overall, the antioxidant system displayed unexpected patterns of response to both radiation doses. At 50 Gy, the activities of POD, CAT, and content of AsA were all increased compared to the control (**Figures 5F, H**), while the activity of SOD decreased (**Figure 5E**). At 200 Gy, all members of the antioxidant system experienced a decrease in activity or content compared to the control.

The direct effects of irradiation on cells were of very short duration (<10^-12^s), and radiolysis was no longer possible during seedling development, so all subsequent ·OH production was generated by the reaction of H_2_O_2_ with ·O_2_^-^. This study found that the rate of ·OH generation was similar at both doses, but the content of H_2_O_2_ and ·O_2_^-^ content were different. ·O_2_^-^ had more time to be catalyzed by SOD to generate H_2_O_2_ in the cell due to its slower diffusion rate and reaction with biomolecules (Riley, 1994; Andrés et al., 2023). Therefore, although the level of ROS at 200 Gy was the same as that at 50 Gy, the enzyme activity and AsA content were significantly reduced, the MDA content was significantly increased, suggesting that high-dose irradiation caused disruption of the plant antioxidant system, resulting in the inability to repair excessive oxidative damage and abnormal plant growth and development. Conversely, at 50 Gy, the antioxidant system of the seedlings was functioning actively, which promotes plant growth and development.

### A general overview of transcriptome

**Figure 6:**
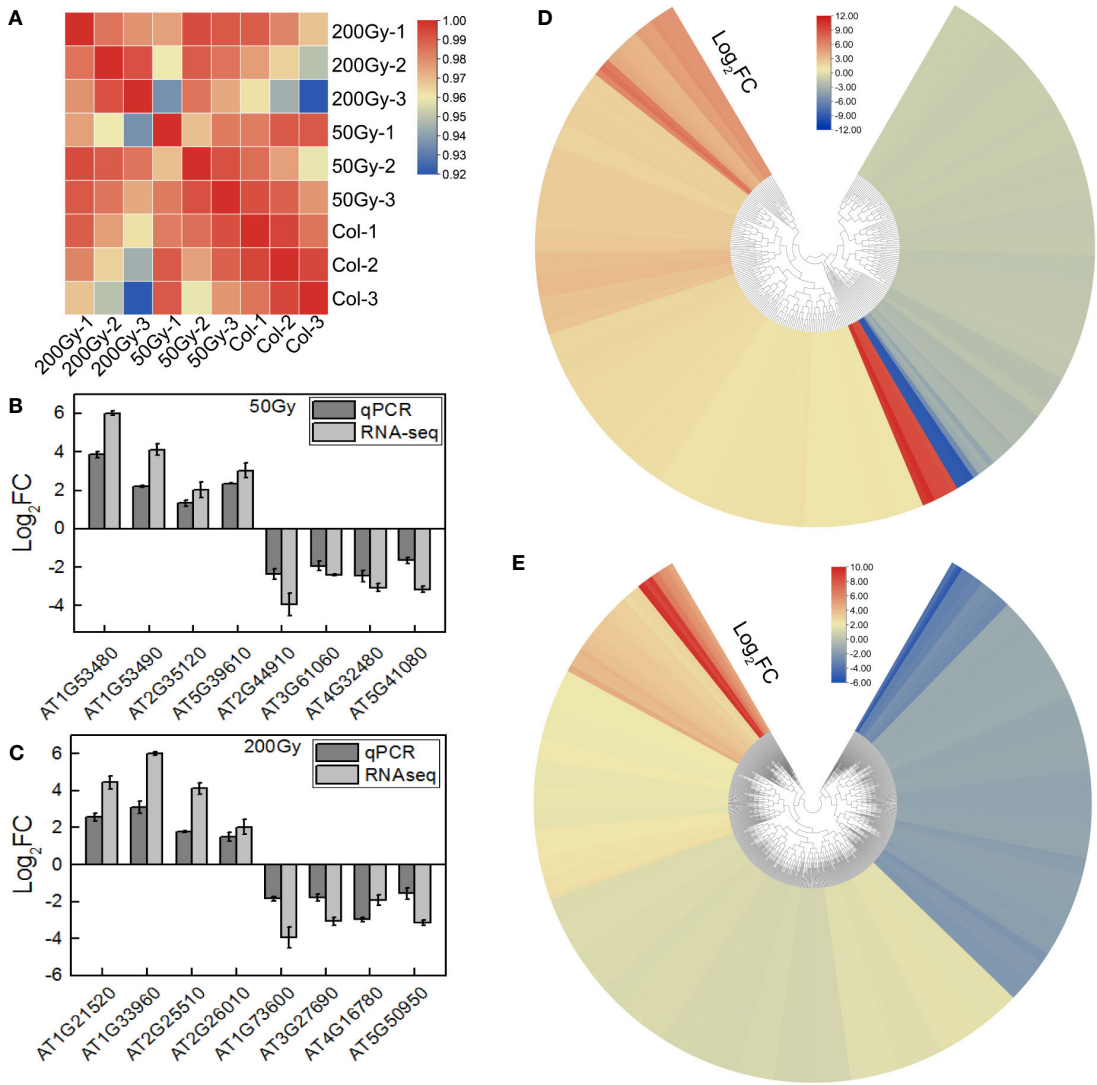
RNA-seq of *Arabidopsis* seedlings from irradiated seeds. **(A)** Heatmap of correlation between samples of RNA-seq; **(B, C)** qRT-PCR verification of part of the RNA-seq results, calculated by 2 ^-ΔΔCt^; **(D, E)** Heatmap of DEGs for 50 Gy and 200 Gy irradiation.

The raw data was filtered to obtain an average of 21.157 million clean reads per sample with a Q30 score of 96.5%. These data were compared to the TAIR reference genome and more than 95% of the reads were mapped successfully (**Supplementary Table S2**). The reproducibility of the data obtained from the same group was high (Pearson correlation >92%) (**Figure 6A**). To validate the RNA-seq, 16 DEGs were randomly selected for qRT-PCR analysis. The qRT-PCR results were consistent with the log_2_ fold change (FC) trend of the RNA-seq data (**Figures 6B, C**), indicating that the RNA-seq data was reliable for further analysis.

**Figure 7:**
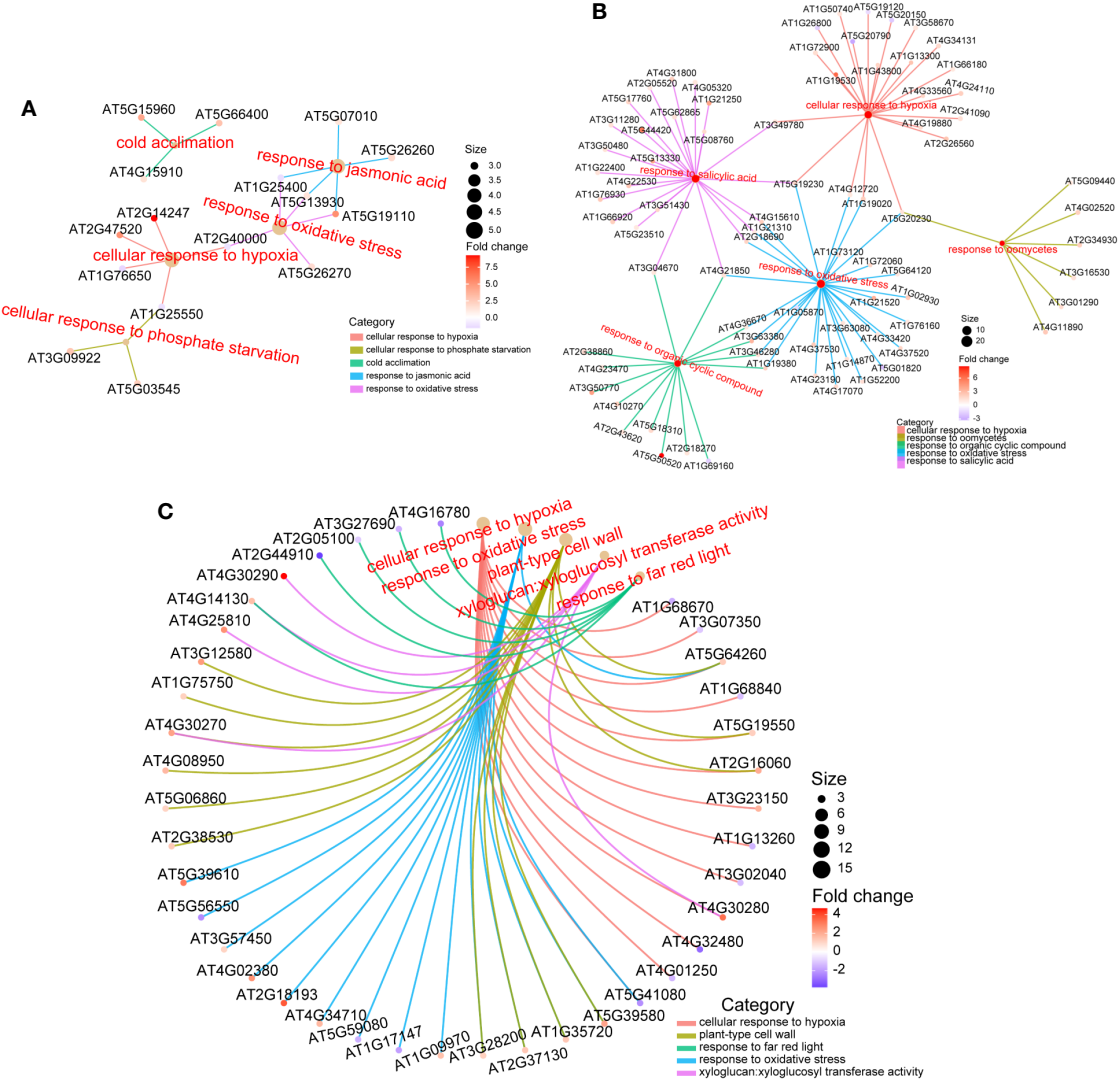
GO enrichment of DEGs. **(A)** for DEGs only in 50 Gy, **(B)** for DEGs only in 200 Gy, and **(C)** for DEGs both in 50 Gy and 200 Gy. Different colored lines indicate enrichment in different term, the color of the prototype symbol indicates the fold change of differences, and the size indicates the ratio of enrichment.

To further explore the biological effects of various doses of ion beams on seedlings, we delved into the sequencing data. The RNA-seq results revealed that 228 DEGs were identified in the 50 Gy which 137 were up-regulated and 91 were down-regulated (**Figure 6D**, **Supplementary Figure S2A**), Meanwhile, 605 DEGs were identified in the 200 Gy, with 395 up-regulated and 210 downregulated (**Figure 6E**, **Supplementary Figure S2B**). Moreover, we compared the two sets of sequencing data and found that 58 genes were only altered in the 50 Gy, 435 genes only in the 200 Gy, with 170 DEGs shared by the two groups (**Supplementary Figure S2C**). Analysis of the DEGs revealed that those specifically expressed in 50 Gy were mainly associated with ‘cold acclimation’, ‘response to jasmonic acid’, ‘response to oxidative stress’, ‘cellular response to phosphate starvation’ and ‘cellular response to hypoxia’. DEGs that were exclusive to 200 Gy were mainly concentrated in ‘cellular response to hypoxia’, ‘response to oxidative stress’, ‘cellular response to salicylic acid’, ‘response to organic cyclic compound’ and ‘response to oomycetes’. It was remarkable that DEGs in both 50 Gy and 200 Gy were localized to ‘response to oxidative stress’, ‘cellular response to hypoxia’, ‘plant-type cell wall’, ‘xyloglucan: xyloglucosyl transferase activity’ and ‘response to far red light’ (**Figure 7**). Although the different doses of irradiation induced the production of DEGs with varying enrichment results, it can be concluded that *Arabidopsis* responds to C-ion beam irradiation mainly through oxidative stress and cell wall dynamic homeostasis-related pathways.

In this study, a total of 170 DEGs were identified between the treatment groups (50 Gy and 200 Gy irradiation) and the control. Among them, more than 25 genes were related to oxidative stress, including *RLK7* (AT1G09970), *OXS3* (AT5G56550) and *ORE1* (AT5G39610). These genes have previously been shown to significantly affect plant oxidizing resistance (Woo et al., 2004; Blanvillain et al., 2009; Pitorre et al., 2010). ROS levels in seedlings were examined in this study and we observed that they were increased regardless of irradiation dose, which may explain the presence of a large number of oxidative stress-related DEGs. In brief, the differential expression of oxidative stress-related genes under different doses of irradiation suggests that they collectively respond to radiation and synergistically resist irradiation damage.

The 50 Gy irradiation treatment produced only 228 DEGs compared with the control, which is roughly 1/3 of 200 Gy produced. There were 58 and 435 DEGs that appeared uniquely in the 50 and 200 Gy irradiation treatments. GO enrichment analysis of the two groups of DEGs mentioned above reveal that in addition to ‘response to oxidative stress’ and ‘ cellular response to hypoxia’, which is conserved in both of the DEGs, genes respond to jasmonic acid signaling were also differently expressed in the 50 Gy DEGs, which may be related with the physiological phenotype of *Arabidopsis* seedlings after low-dose irradiation.

In addition, among the unique differentially expressed genes (DEGs) associated with 200 Gy irradiation, while there are instances of ‘response to oxidative stress’ and ‘cellular response to hypoxia,’ a substantial number of genes are also enriched in the category of ‘response to salicylic acid.’ This observation may suggest that the distinct phenotypes observed at 50 Gy and 200 Gy are a result of radiation-induced variations in ROS and hormone response.

### Transcriptional investigation of the growth promotion induced by 50 Gy ^12^C^6+^ ion beam irradiation

The seeds exposed to 50 Gy irradiation had increased the development rate at the seedling stage. Through GO enrichment analysis, we found that the DEGs at 50 Gy were mainly associated with response to oxidative stress, cold acclimation, cellular response to hypoxia and response to jasmonic acid (**Figure 7A**).

The ROS-related genes that were upregulated under the 50 Gy irradiation treatment, such as peroxidase (AT5G39580, AT3G28200), glutathione s-transferase (AT1G17170), and ascorbate oxidase (AT5G21105), were identified in this study. ROS have been found to play a crucial role in plants and may regulate plant growth responses to radiation (Huang et al., 2019; Tan et al., 2023). For instance, Overmyer et al. (2005) found that the expression of AT5G39580 was reduced in ozone-exposed environments. Pavet et al. (2005) demonstrated that the expression of AT3G28200 was increased during redox response, suggesting it may be involved in plant resistance to oxidase stress. Additionally, Rahantaniaina et al. (2013) reported that glutathione S-transferase (AT1G17170) converts GSH to GSSG by exogenous H_2_O_2_ application. Yamamoto et al. (2005) also found that the ascorbic acid oxidase (AT5G21105) mutant accumulated more AsA than the wild type under salt stress, confirming its importance in salt stress-induced oxidative stress.

Previous studies have demonstrated that chalcone synthase *TT4* (AT5G13930) is highly expressed after UV-B irradiation, resulting in a significant production of flavonoids and thus increased resistance (Nakabayashi et al., 2014; Zhou et al., 2023). Song et al. (2008) found that overexpression of ATG25400 resulted in increased resistance to oxidative stress. Our study also identified changes in the expression of several WRKY family members, with *WRKY45* being uniquely upregulated at both irradiation doses. Chavan et al. (2022) applied exogenous dehydroascorbate (DHA) to the foliar surface of rice, which induced a substantial increase in the ROS content of the whole rice plant as well as a significant increase in *WRKY45* expression, suggesting that *WRKY45* is also involved in ROS signaling regulation. Our RNA-seq and ROS analysis results suggests that seedlings may use the ROS produced by irradiation as signaling molecules to accelerate various developmental and metabolic process, thereby improving the development rate of plants at the seedling stage.

Jasmonic acid (JA) is involved in a wide range of development activities and plays a major role in plant stress tolerance (Ghorbel et al., 2021; Li et al., 2021). RNA-seq analysis result indicated that the expression of certain JA related WRKY family members (*WRKY33* and *WRKY45*) and genes such as AT5G07010 and AT5G26260 was upregulated at the 50 Gy dose, which may increase the stress tolerance of seedlings. Noteworthily, the findings were in accordance with Ueda et al. (2015) research, who found that knockdown of *WRKY45* in rice significantly affected the expression of downstream JA- and SA-related genes, thereby affecting the plant resistance to oxidative environments.

Our previous research determined that 50 Gy irradiation increased seedlings tolerance to cold environment (Wang et al., 2018b). GO enrichment analysis in this study partially confirmed this finding, suggesting that multiple genes may be involved in the cold acclimation, which could be beneficial for further research on improving cold tolerance in plants.

Moreover, genes related to xyloglucan were also enriched. Members of the Xylan or XTH family are known to be involved in cell wall expansion and reorganization (Rose et al., 2002; Stratilová et al., 2020; De Caroli et al., 2021). For instance, overexpression of *XTH24* (AT4G30270), *XTH18* (AT4G30280) or *XTH19* (AT4G30290) has been shown to cause hypocotyl or root elongation in *Arabidopsis* (Miedes et al., 2013; Lee et al., 2018; Dhar et al., 2022), which is similar to what we observed in early seedling development (**Figure 4**). Therefore, root elongation induced by 50 Gy irradiation may be associated with the up-regulation of some XTH family genes.

### Transcriptional investigation of the growth inhibition by 200 Gy ^12^C^6+^ ion beam irradiation

GO enrichment analysis of DEGs after 200 Gy ^12^C^6+^ ion beam irradiation revealed that the expression of genes related to oxidative stress were altered, which is in accordance with the ROS determination as described above (**Figure 4**, **7B**). One study was conducted to investigate the effects of ion beam-induced ROS production on organisms, founding that high-dose ion beam irradiation leads to the burst of ROS (Matsumoto et al., 2021). In our study, the expression of eight peroxidase family members was up-regulated under 200 Gy irradiation, but the activity of POD and CAT decreased, which may partially account for the increase in H_2_O_2_ and ·O_2_^-^ in *Arabidopsis* seedlings. In addition, six genes related to glutathione metabolism were also up-regulated, which was consistent with the decrease in AsA content and SOD activity. Therefore, it is difficult for the antioxidant system of the plant to maintain its balance in a high dose generated ROS environment, resulting in the plant’s inability to self-regulate oxidative stress. Taken together, excessive irradiation dose induced the accumulation of more ROS in *Arabidopsis* seedlings, which led to the disruption of the antioxidant system and the surge of lipid peroxidation in the cell membrane, and eventually resulted in seedling developmental disorders.

Salicylic acid has been extensively studied in plants against abiotic stresses and is particularly important in oxidative stress defense processes (Radwan, 2012; Herrera-Vásquez et al., 2015). Our research revealed that more than 20 DEGs were associated with SA response, suggesting that the SA metabolic pathway plays a significant role in defense against high-dose C-ion beam irradiation. Additionally, the GO enrichment results for other stresses, such as response to organic cyclic compound and response to oomycetes, were also observed in the 200 Gy heavy ion beam irradiation, suggesting that the defense responses induced by 200 Gy of heavy ion beam irradiation is somewhat similar to those induced by organic cyclic compound and oomycetes.

The expression of XTH family members (*XTH15*, *XTH18*, *XTH19*, *XTH23*, *XTH24*, *XTH30*) was increased under 200 Gy irradiation, which is similar to the pattern under 50 Gy irradiation. Considering the contrastive effects of 50 Gy and 200 Gy treatments on the growth of *Arabidopsis*, it is likely that the inhibition of *Arabidopsis* growth at 200 Gy may also be caused by changes in other genes or pathways besides the ROS pathway.

As noted in our text, DEGs in 200 Gy are mainly enriched for cell wall homeostasis as well as against oxidative stress (**Figure 7**). To explain that the growth of 200 Gy seedlings was inhibited but the number of up-regulated genes was more than that of down-regulated genes, we put the DEGs list on display (**Supplementary Table S4**). It can be seen that 6 genes belong to plant defensin (PDF) family, 7 genes belong to peroxidase, 6 genes belong to xyloglucan endotransglucosylase/hydrolase family (XTH), 4 genes belong to WRKY family of transcription factors, etc.

Previous research indicated that proteins of the PDF family inhibit root development (Allen et al., 2008), while members of the XTH family participate in the reorganization and stabilization processes of plant cell walls, thereby influencing root development (Miedes et al., 2013). Peroxidase is identified as a crucial component in the plant’s response to oxidative stress (Kim et al., 2012; Ling et al., 2013), and several members of the WRKY transcription factor family exhibit upregulated expression in response to ionizing radiation (Nagata et al., 2005; Goh et al., 2014). These pieces of evidence elucidate the correlation between our RNA-seq results and phenotypic observations.

## Conclusion

Low-dose heavy ion beam irradiation induces ROS production in plants, thereby accelerating seedling growth, while high-dose irradiation leads to the accumulation of excess ROS and thereby severely inhibits plant growth. Monte Carlo simulation results indicated that higher doses of irradiation induced more complex damage patterns compared to the lower doses. Both doses have little effect on the structural organic components but activate a large number of genes in response to stress, forming a response system that is centered on oxidative stress signals and other stress response pathways. In addition, plant hormones such as SA and JA may work together to respond to radiation.

## Data availability statement

The original contributions presented in the study are publicly available. This data can be found here: https://ngdc.cncb.ac.cn/bioproject/browse/; PRJCA021264.

## Author contributions

YY: Writing – original draft, Investigation, Visualization. DC: Writing – review & editing. QC: Investigation, Writing – review & editing. HX: Investigation, Writing – review & editing. PG: Writing – review & editing. HZ: Investigation, Writing – review & editing. TJ: Investigation, Writing – review & editing. XW: Investigation, Writing – review & editing. LW: Investigation, Writing – review & editing. HS: Writing – review & editing.
