# Supplementary material for: Reactive oxygen species may be involved in the distinctive biological effects of different doses of 12C6+ ion beams on Arabidopsis
Source: Front Plant Sci. 2024 Jan 19;14:1337640. doi: 10.3389/fpls.2023.1337640 (PMC10835405; doi:10.3389/fpls.2023.1337640)
Supplement: Supplementary file 2 [file DataSheet_1.docx]

**Table S1.** Content ratio of main components in *Arabidopsis* seeds. (Krebbers et al., 1988; Pang et al., 1988; Western et al., 2000; Baud et al., 2002; Monsalve et al., 2003; O'Neill et al., 2003; Gruis et al., 2004; Moïse et al., 2005; Li et al., 2006; Gardarin et al., 2010; Kreitschitz et al., 2018; Jayawardhane et al., 2020)

|  | C | H | O | N | P | S |
| --- | --- | --- | --- | --- | --- | --- |
| - Content ratio | 0.269187 | 0.568268 | 0.126684 | 0.033934 | 0.000165 | 0.001762 |

**Table S2.** Filtering and comparison of transcriptome sequencing data

| Sample | Total Clean Reads (Mb) | Clean Reads Q30(%) | Total Mapping Ratio |
| --- | --- | --- | --- |
| Zcol-1 | 21.18 | 96.73% | 95.33% |
| Zcol-2 | 21.21 | 96.78% | 96.43% |
| Zcol-3 | 21.1 | 96.49% | 96.35%  95.58% |
| 50-1 | 21.2 | 96.75% |  |
| 50-2 | 21.26 | 96.83% | 95.82% |
| 50-3 | 21.04 | 96.66% | 96.33% |
| 200-1 | 21.14 | 96.61% | 96.86% |
| 200-2 | 21.24 | 96.73% | 96.74% |
| 200-3 | 21.05 | 96.72% | 96.37% |

**Table S3.** Primers used in this study

| Gene | primers |
| --- | --- |
| AT1G53480 | F: CAAATCGGTTTATCAATCTC |
|  | R: TCGACTTAAAACTCTTTGAT |
| AT1G53490 | F: ATTCACCCAATGACACGA |
|  | R: GGCTTAGGACCGAAATAG |
| AT2G35120 | F: CCCCTGGATTGGTGAACT |
|  | R: TTGGAGAAGGATGCTGGA |
| AT5G39610 | F: GAGGCATCAAGAATCGTC |
|  | R: ACCAATGGCAGTAGCAGA |
| AT2G44910 | F: CAGAATAACAATAGCCATCC |
|  | R: AAACCTCTTAGAAATGACCC |
| AT3G61060 | F: TCTATTTCTTCAAAGGCACT |
|  | R: CTCCTCCTACTTCAAACCAC |
| AT4G32480 | F: CGACGAATCCGAAGTTGA |
|  | R: AACGCTTCCTTAGTTGCTTG |
| AT5G41080 | F: TGATTTGGATGATTCTCTTT |
|  | R: GGAAACTTGAGAAGATTACT |
| AT1G21520 | F: TCGTGGTGTTTAAAGATATT |
|  | R: AGATATTAAGCCCATCATTC |
| AT1G33960 | F: TACACGCTGTGCTCTTAG |
|  | R: ATACCGTCATCTTCCAATA |
| AT2G25510 | F: AGGAATCCCAGAAACAAG |
|  | R: GAGGAGGAGATAAATGAAGA |
| AT2G26010 | F: ACAAGTGTATCTGTTACTTC |
|  | R: GCATGTCATAATAAAGTCAC |
| AT1G73600 | F: ACACTACCGTGAACCTAAA |
|  | R: CCCTATCATTATCCGAACT |
| AT3G27690 | F: ACCGTCAAGTCTACTCCTCA |
|  | R: CCCAACATTGCCCATCTA |
| AT4G16780 | F: AGTTCTTACGGAGATGCTG |
|  | R: GTCAAAGTAGTGGGTGGG |
| AT5G50950 | F: GTCCTTCGAGTTTAAAGATA |
|  | R: TACATCAAACCCTTTCTTAG |


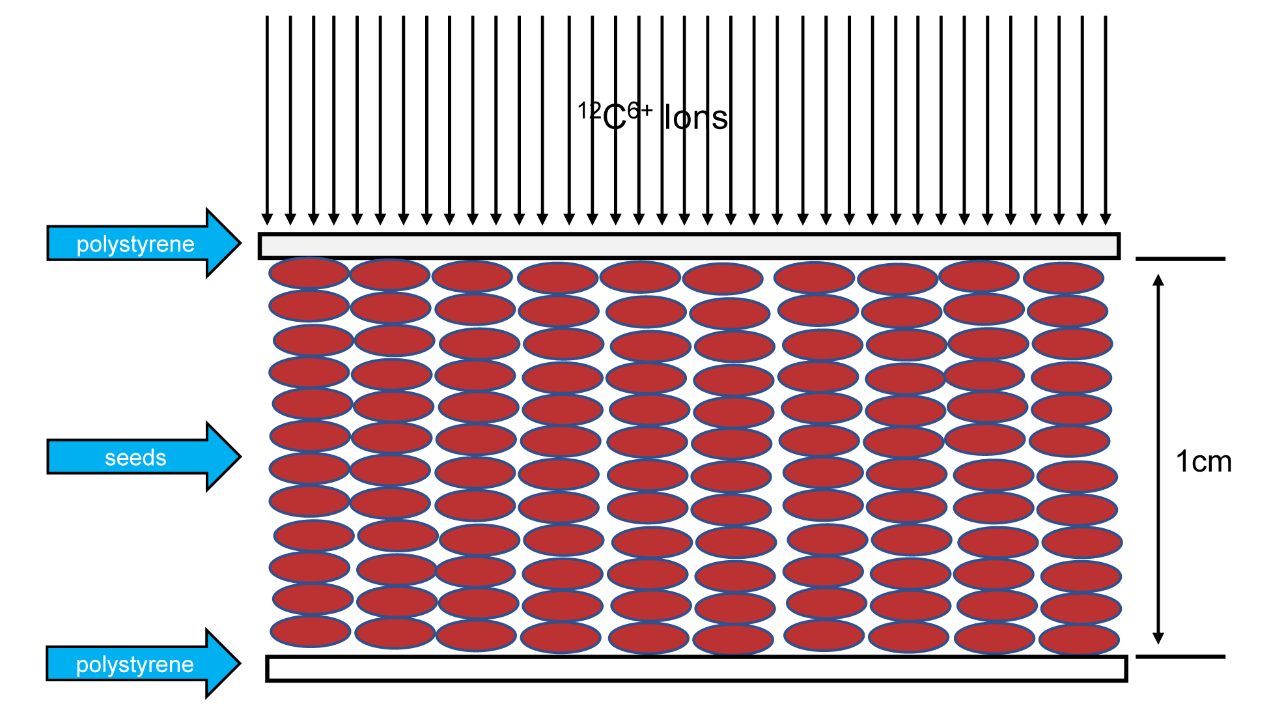


**Fig S1.** Schematic diagram of a simple model of a target. The model is composed of petri dishes filled with *Arabidopsis* seeds made up of polystyrene plastic, and the irradiated ions are injected from a direction perpendicular to the dish. Red ovals represent *Arabidopsis* seeds


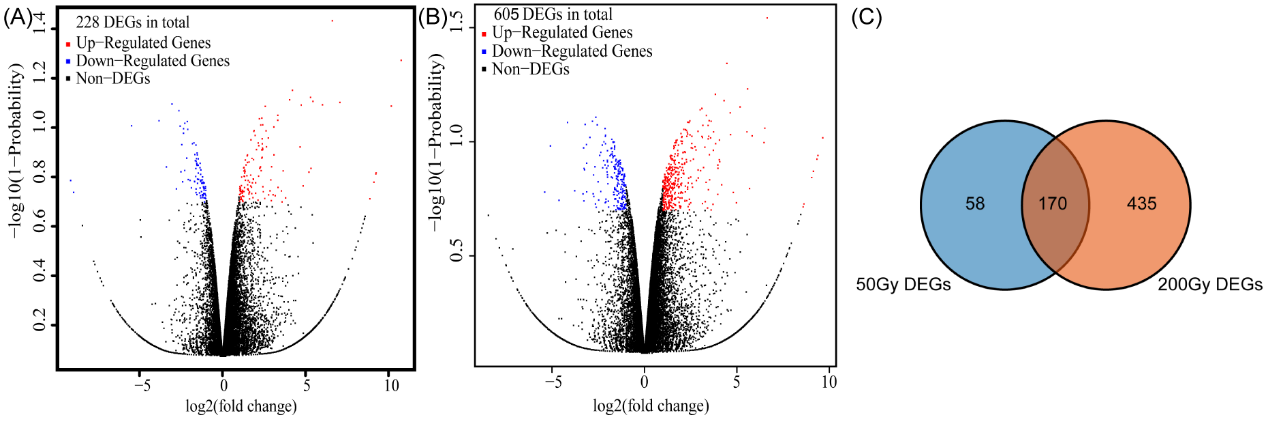


**Fig. S2** 50Gy and 200Gy DEGs. (a) and (b) show the number of DEGs up- and down-regulated at 50 and 200 Gy, respectively. Red spots indicate up-regulated genes, blue spots indicate down-regulated genes, and black indicates no-change genes. (c) shows the genes that are co-differentiated between the two sets of sequencing as well as the genes that are unique to each.


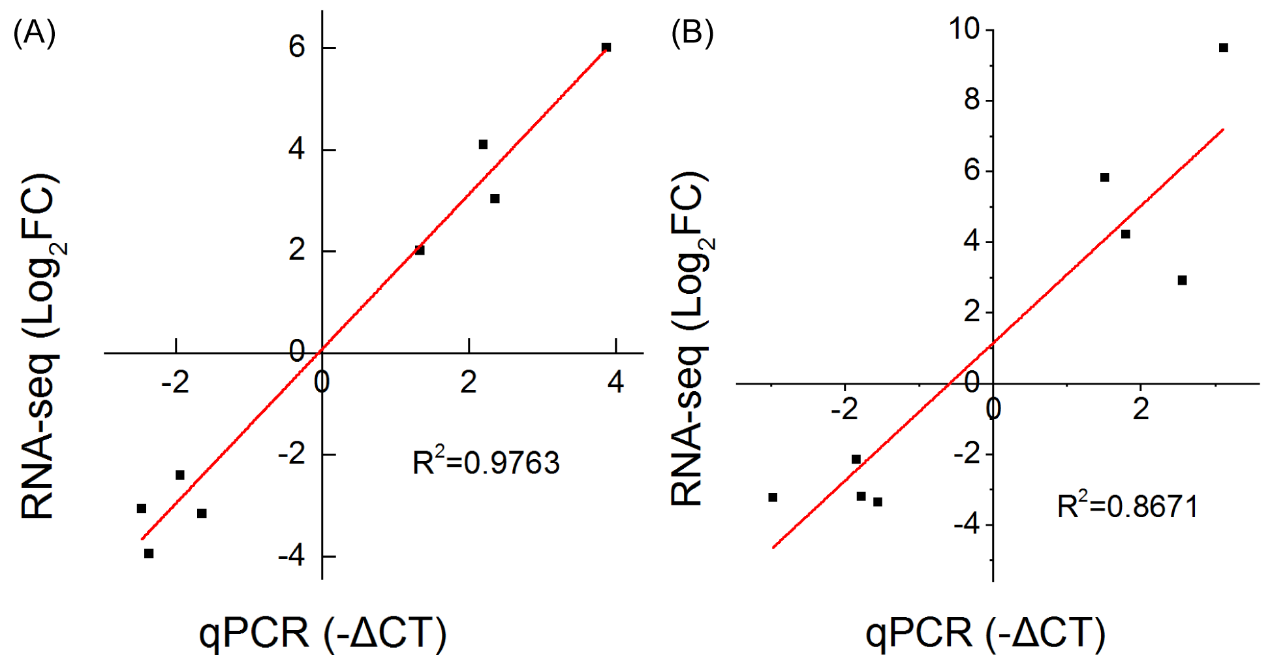


**Fig. S3** Correlation analysis of RNA-seq and qPCR results. (A) and (B) show the correspondence between 50Gy and 200Gy RNA-seq and qPCR results, respectively, with R^2^ > 0.85.

**Reference**

Baud, S., Boutin, J.-P., Miquel, M., Lepiniec, L., and Rochat, C. (2002). An integrated overview of seed development in *Arabidopsis thaliana* ecotype WS. *Plant Physiol Biochem* 40(2)**,** 151-160.

Gardarin, A., Dürr, C., Mannino, M.R., Busset, H., and Colbach, N. (2010). Seed mortality in the soil is related to seed coat thickness. *Seed Sci Res* 20(4)**,** 243-256. doi: 10.1017/s0960258510000255.

Gruis, D., Schulze, J., and Jung, R. (2004). Storage protein accumulation in the absence of the vacuolar processing enzyme family of cysteine proteases. *Plant Cell* 16(1)**,** 270-290. doi: 10.1105/tpc.016378.

Jayawardhane, K.N., Singer, S.D., Ozga, J.A., Rizvi, S.M., Weselake, R.J., and Chen, G. (2020). Seed-specific down-regulation of *Arabidopsis* CELLULOSE SYNTHASE 1 or 9 reduces seed cellulose content and differentially affects carbon partitioning. *Plant Cell Rep* 39(7)**,** 953-969. doi: 10.1007/s00299-020-02541-z.

Krebbers, E., Herdies, L., De Clercq, A., Seurinck, J., Leemans, J., Van Damme, J., et al. (1988). Determination of the processing sites of an *Arabidopsis* 2S albumin and characterization of the complete gene family. *Plant Physiol* 87(4)**,** 859-866.

Kreitschitz, A., and Gorb, S.N. (2018). The micro- and nanoscale spatial architecture of the seed mucilage-Comparative study of selected plant species. *PLoS One* 13(7)**,** e0200522. doi: 10.1371/journal.pone.0200522.

Li, Y., Beisson, F., Pollard, M., and Ohlrogge, J. (2006). Oil content of *Arabidopsis* seeds: the influence of seed anatomy, light and plant-to-plant variation. *Phytochemistry* 67(9)**,** 904-915.

Moïse, J.A., Han, S., Gudynaitę-Savitch, L., Johnson, D.A., and Miki, B.L.A. (2005). Seed coats: Structure, development, composition, and biotechnology. *In Vitro Cell Dev Biol Plant* 41(5)**,** 620-644. doi: 10.1079/ivp2005686.

Monsalve, R.I., Villalba, M., Rico, M., Shewry, P.R., and Rodríguez, R. (2003). The 2S albumin proteins. *Plant food allergens***,** 42-56.

O'Neill, C.M., Gill, S., Hobbs, D., Morgan, C., and Bancroft, I. (2003). Natural variation for seed oil composition in *Arabidopsis thaliana*. *Phytochemistry* 64(6)**,** 1077-1090. doi: 10.1016/s0031-9422(03)00351-0.

Pang, P.P., Pruitt, R.E., and Meyerowitz, E.M. (1988). Molecular cloning, genomic organization, expression and evolution of 12S seed storage protein genes of *Arabidopsis thaliana*. *Plant Mol Biol* 11**,** 805-820.

Western, T.L., Skinner, D.J., and Haughn, G.W. (2000). Differentiation of mucilage secretory cells of the *Arabidopsis* seed coat. *Plant Physiol* 122(2)**,** 345-356.
